# Abstracts of the 2007 Young Invesigators Symposium

**DOI:** 10.1120/jacmp.v8i3.2714

**Published:** 2007-07-23

## Effects of external beam radiation in FDG small animal PET

A. Kesner, V. Lau, M. Speiser, W. Hsuehm, N. Agazaryan, J. DeMarco and D. Silverman

David Geffen School of Medicine, University of California, Los Angeles, CA, USA.

## Objectives

FDG PET is emerging as a tool in radiation therapy for target delineation, and has potential in treatment monitoring. In our experiment we examined the time‐course of FDG uptake in mice in a variety of normal tissues during the 2‐month period following external beam irradiation.

## Methods

Four mice received 12 Gy of external beam radiation in a single fraction to the left half of the body. Small animal PET scans were acquired 1 hr after i.v. administration of 7.5 MBq (0.2 mCi) FDG. Scans were acquired at 0 (pre‐radiation), 1, 2, 3, 4, 5, 8, 12, 19, 24, and 38 days following irradiation. FDG activity in various tissues was compared between irradiated and non‐irradiated body halves before and at each time‐point after irradiation.

## Results

Radiation had a significant impact on FDG uptake in previously healthy tissues, and time‐course of effects differed dramatically in different types of tissues. For example, liver tissue demonstrated increased FDG uptake peaking on day 8, with the mean left to right uptake ratio averaged over days 3–12 increasing 52% over baseline values (p<0.001); in contrast, femoral bone marrow demonstrated decreased FDG uptake over days 2–8, with an uptake ratio decreasing 26% below baseline values (p=0.005). Significant effects of radiation were also seen in lungs and brain.

## Conclusions

We have observed diverse effects of radiation on FDG uptake in the weeks following radiation in several tissues. Further characterization of these kinds of effects may help integrate PET usage into the field of radiation therapy.

## Surviving fraction $\left({\text{SF}}_{2}\right)$ is the dominant predictor of tumor control probability (TCP) in high grade glioma (HGG) treated with intensity modulated radiation therapy (IMRT)

Kimberly B. Prescott, Maria T. Vlachaki, and Salahuddin Ahmad

Oklahoma University Health Sciences Center, Wayne School of Medicine

## Objective

Predicting treatment responses using the TCP model in HGG is limited by uncertainties of α/β, number of clonogenic cells in tumor (CCT) and ${\text{SF}}_{2}$. As HGGs have a higher density of clonogenic cells in the tumor center, a mathematical model of spatial distribution of clonogenic cells was developed to determine CCT. TCP was calculated using various combinations of α/β and ${\text{SF}}_{2}$.

## Materials and Methods

IMRT plans for twenty patients were generated. The prescribed dose was 59.4 Gy in 33 fractions. Clonogenic cells were assumed to be distributed in three spherical shells or regions 1, 2 and 3 with progressively decreasing cell density from 10^5^ cells/cm^3^ in region 1 to 10^2^ cells/cm^3^ in region 3. Using the Niemierko method of Equivalent Uniform Dose (EUD) and Munro‐Gilbert hypothesis, TCP was calculated for α/β of 3, 5, 8, 10 and ${\text{SF}}_{2}$ of 0.3, 0.4, 0.5, 0.6, 0.7 and 0.8.

## Results

The average TCP was 100% for ${\text{SF}}_{2}<0.4$ and zero for ${\text{SF}}_{2}>0.7$. For ${\text{SF}}_{2}$ of 0.5 and 0.6, TCP was above 99.6% and 64.1% respectively. Decreasing α/β values from 10 to 3 resulted in further decreases in TCP irrespective of ${\text{SF}}_{2}$ and region 2 was found to have the lowest TCP when compared to regions 1 and 3.

## Conclusions

TCP modeling predicts that ${\text{SF}}_{2}$ is the dominant predictor of radioresistance. Relating clinical prognostic indicators to ${\text{SF}}_{2}$, CCT and α/β and identifying spatial tumor factors relating to TCP will improve our ability to model response to therapy in HGG.

## Temporal Re‐Binning for Slow‐Acquisition Megavoltage CT Scans

S. Scott Outten, Chester Ramsey, and Rebecca Siebert

Thompson Cancer Survival Center, Knoxville TN

### Purpose

Images obtained from slow‐acquisition scans often feature motion artifacts as a result of respiration, the majority of which occurs in the superior‐inferior (S/I) direction. The purpose of this work was to correct for S/I motion temporal re‐binning.

### Method and Materials

An ACR accreditation Phantom and an anthropomorphic thorax phantom were moved 3‐cm through a 5‐mm MVCT slice thickness over periods of 2, 4 and 6 seconds. The Scan data was acquired with 5 rotations of the gantry and a stationary couch. Motion correction was performed by extracting projections occurring within specific respiration amplitudes, correcting for missing projections, and then reconstructing the CT images. Several patients with lung tumors without mediastinal involvement were also scanned. Motion was measured using the Varian respiratory gating system.

### Results

Re‐binned images acquired with the ACR phantom showed considerable improvement with motion correction using temporal re‐binning over the uncorrected images. In comparison, images acquired with the thorax phantom featured less improvement than the ACR phantom due to variations within the phantom's structure. Each phantom featured noticeable improvement over each period. Improvements were also observed in patient scans; however a 4D‐CT scan was unavailable for verification.

## Conclusion

Temporal re‐binning can successfully reduce S/I motion artifacts in reconstructed MVCT images, and may help provide a method for developing four dimensional image guidance systems. Patient scanning requires diagnostic 4D‐CT scans to verify the accuracy of the scan.

Conflict of Interest (only if applicable): This research is supported by TomoTherapy, Inc.

## Ramipril reduces spinal cord injury after radiation exposure

Sanath Kumar, G. Zhu, S.L. Brown, A. Kolozsvary, Fang‐Fang Yin and J.H. Kim

Department of Radiation Oncology, Henry Ford Hospital, Detroit, MI 48202

### Purpose

Mitigation of radiation injury to rat spinal cord by Ramipril, an angiotensin converting enzyme inhibitor, given after an x‐ray exposure was studied.

### Materials and Methods

Groups of rats (n=18 total, 3 groups) were either untreated or irradiated to the cervical spinal cord with 22 Gy using 6 MeV x‐rays. Irradiated rats received ramipril (1.5 mg/kg/day) 24 hours after radiation, continuing for the study duration, or received radiation alone. All of the animals were monitored for signs of paralysis in the limbs. At seven months, rats were sacrificed and examined for histological lesions associated with radiation damage, such as necrosis or demyelization using Luxol Fast Blue staining. Demyelination using Relative Optical Density (ROD) was quantified using computer based image analysis system.

### Results

Within 7 months following up, partial paralysis of limbs developed in two animals that received radiation alone and one animal in radiation plus ramipril treated group. All the animals had some evidence of demyelization with a mean ROD of 0.119 for the radiation alone group compared to a ROD of 0.143 for the ramipril treated group (T‐test p=0.00002). In comparison, ROD in untreated spinal cord was 0.254, significantly higher indicating that ramipril offered some mitigation of radiation injury, but did not reduce damage to the level of untreated control.

### Conclusion

Ramipril given after radiation injury offers a small but statistically significant reduction in demyelization of spinal cord induced by radiation.

## Tools for Identifying Head and Neck Setup Errors in Image Guided Radiation Therapy

Ben Robison, Chester Ramsey, and Rebecca Siebert

Thompson Cancer Survival Center, Knoxville TN

### Purpose

The primary objective of this study was to develop a novel tool using Kernel Classification that could be used to assist in identifying patients with unacceptable random and systematic setup errors.

### Method and Materials

Inter‐Fraction motion was analyzed for 30 Head and Neck (H&N) patients that were positioned for treatment using megavoltage CT (*MVCT*) images acquired on a helical tomotherapy system. Adjustments in the AP, SI, LAT and Roll planes were made using the MVCT images to ensure correct target alignment.

### Results

Thirty H&N patients were MVCT imaged prior to treatment delivery for a total of 992 imaging sessions. 17% of the patients were incorrectly positioned on the headrest, 13% were incorrectly positioned inside the aquaplastic mask, 10% had changes in the mask shape, 17% had weight loss, and 27% had a measurable radiation response. The kernel classification technique correctly identified 29 out of the 30 H&N patients as either having a normal or problematic setup using the respective shift data sets. These classifications were made using only the positional shift values from the first 14 treatments.

### Conclusion

This study demonstrated that the kernel regression classification method was able to correctly identify the cause behind IGRT positioning problems for individual H&N patients. Since this technique is fully automated, it could easily be implemented on the IGRT operator's computer to determine the reason a patient is having positioning errors early in their treatment, and also to give more information about whether a second opinion is needed before treating the patient.

## The Role of Secondary Particles in Proton Therapy

Youssef Charara, Chester Ramsey, and Rebecca Siebert

Thompson Cancer Survival Center, Knoxville TN

### Purpose

The purpose of this work was to evaluate the contribution of energy deposition from primaryproton particles versus the contribution of secondary particles.

### Method and Materials

Contributions of energy deposition from primary protons versus secondary particles were calculated using HETC‐HEDS, which simulates particle cascades using Monte Carlo to compute the trajectories of the primary particles and all secondary particles produced in nuclear collisions. Simulations were performed using incident proton beams between 90 and 200 MeV/Nucleon in 5 MeV/Nucleon increments incident on 10 g/cm^2^ of A‐150 Tissue Equivalent Plastic.

### Results

In proton therapy, secondary particles contributed approximately 50% of the total energy deposited. While it is well known that spallation can occur during proton therapy, the distributions of the spallation products after the Bragg peak are often ignored in treatment planning calculations. For a 90 MeV proton beam, only 29% of the charged particles just beyond the Bragg Peak are protons. The remaining 71% of the charged particles after the Bragg peak are heavy nuclei ranging in mass from helium (21%) to Carbon (8%).

### Conclusion

For proton therapy beams, Monte Carlo simulations indicate that about 50% of total energy deposition originates from secondary particles. Furthermore, a significant fraction of the fluence beyond the Bragg Peak for proton comes from secondary particles. Given that critical structures are often located near the Bragg peak it is vital that the effects of these particles be taken into consideration.

## The Importance of Plan‐to‐Film Registration in Intensity Modulated Radiation Therapy Quality Assurance

Carley Harris, Chester Ramsey, and Rebecca Siebert

Thompson Cancer Survival Center, Knoxville TN

### Purpose

The purpose of this study was to evaluate the advantages and disadvantages of manual and fiducial‐based plan‐to‐film registration techniques.

### Method and Materials

Ten patient plans (*4 Head & Neck and 6 Prostate*) were selected for this IMRT plan‐to‐film registration study. IMRT dose measurements were obtained in both axial and coronal planes using radiographic and radiochromic film. The calculated and measured dose distributions were registered with one another using the RIT113 dosimetry software. Manual registration was performed by visual selection of four points in common on the dose and film images. Fiducial‐based registration was performed using the “Template” tool in RIT113 and marks placed on the films prior to irradiation.

### Results

A total of 40 IMRT QA films were irradiated and analyzed. The manual registration technique was highly susceptible to inter‐ and intra‐user variations. In fiducial‐based registration, the film slipped during phantom assembly and compression on multiple occasions. This resulted in multiple fiducial marks in 35% of the films, and rotation of the film in 30% of the irradiations. Another problem encountered with fiducial‐based registration was light‐leakage after EDR film was marked, which resulted in large fiducial marks in 55% of the films.

### Conclusion

In order to perform IMRT QA with film, the measured dose distributions must be aligned (*or registered*) correctly with the expected dose distributions from the treatment planning system. Manual registrations were susceptible to inter‐ and intra‐user variations, while fiducial‐based registrations can have incorrect placement of the fiducials on the film.

Conflict of Interest: Research supported RIT

## High Contrast Imaging With Orthogonal Bremsstrahlung Beams: A Novel Technique.

Arman Sarfehnia, Keyvan Jabbari, Jan Seuntjens, and Ervin B. Podgorsak

McGill University, Montreal, Quebec, Canada

Since portal images are created by megavoltage, forward‐directed bremsstrahlung beams, their image quality is inferior to that of images produced by kilovoltage beams. We evaluated the suitability of orthogonal bremsstrahlung beams produced by megavoltage electrons for high contrast imaging. A 10 MeV electron beam emerging from the research port of a Varian Clinac‐18 linac was made to strike targets of carbon, aluminum and copper. Percentage depth dose and attenuation measurements of orthogonal bremsstrahlung beams produced in these targets revealed that the effective energy of the orthogonal beam is always lower than that of the forward beam with the reduction being more pronounced for lower atomic number targets. At 100 cm SSD, the mean energy of the orthogonal beam from a carbon target is 6 times lower than that of the forward beam and amounts to 217 keV as opposed to 1.3 MeV measured in the forward direction. In the orthogonal direction, the dose rate was measured to be 0.30 cGy/min at 50 cm for an aluminum target. In the same direction, the photon energy fluence yield was three orders of magnitude smaller for the carbon target relative to the copper target. Images of contrast objects were also taken with several target materials, and it was qualitatively determined that orthogonal bremsstrahlung beams produced by megavoltage electrons striking low atomic number targets yield images with a significantly higher contrast than do forward bremsstrahlung beams.

## Dosimetric Effects of Gantry Angle Misalignment during Helical Tomotherapy Treatment

Fan‐Chi Su, Cheng Yu Shi, Martin Fuss, and Niko Papanikolaou

University of Texas Health Science Center at San Antonio, San Antonio, TX; Cancer Therapy and Research Center, San Antonio, TX; Oregon Health and Science University, Portland, OR

### Purpose

To investigate the dosimetric effect of gantry angle misalignment on the target volume and critical organs during helical tomotherapy treatment.

### Materials and methods

Five prostate cases were chosen to calculate the effects of gantry angle deviation on both patient‐specific delivery quality assurance (DQA) and treatment plans. For DQA plans, the phantom rotated for $+/-\;5^{\circ }$ from the preset position to simulate the gantry angle deviation during tomotherapy. Point doses at 5 mm below the isocenter and the dose distribution for each gantry angle were measured and reconstructed respectively. For actual patient treatment plans, the same effect was simulated by adjusting the automatic roll correction for $+/-\;5^{\circ }$. The TomoTherapy Planned Adaptive software was used, and variations of DVHs were evaluated for both PTV and critical organs.

### Results

There is no significant difference among point dose measurements for gantry rotation within $+/-\;5^{\circ }$ for DQA plans. Isodose line shifts could be observed for gantry rotations larger than 2°. Discrepancies are also found among DVHs of the PTV in the cases when gantry angle misalignment is larger than 2°. As for DVHs of either bladder or rectum under different gantry rotations, no significant differences are observed among these DVH curves.

### Conclusion

Point dose measurements alone cannot reveal this dosimetric deviation. Significant discrepancies were found in dose distributions of the DQA plans and DVHs of PTVs when gantry angle variation was larger than 2°. Specific quality assurance procedures should be made to assure that the gantry angle accuracy is within $+/-\;2^{\circ }$.

## Comparison of transabdominal ultrasound and electromagnetic transponders for prostate localization: clinical experience in a large patient population

Ryan D. Foster, Indrin J. Chetty, Haisen Li, Charles A. Enke, Timothy D. Solberg, Twyla Willoughby, and Patrick Kupelian

Department of Radiation Oncology, University of Nebraska Medical Center, Omaha, NE; Department of Radiation Oncology, MD Anderson Cancer Center, Orlando, FL

### Purpose/Objective(s)

The aim of this study is to compare two methodologies of prostate localization in a large cohort of patients.

### Materials/Methods

Daily prostate localization using BAT has been performed at our institution since 2000. More recently, a technology using electromagnetic transponders implanted within the prostate was introduced into our clinic (Calypso®). With each technology, patients were localized initially using skin marks. Localization error distributions were determined from offsets between the initial setup positions and those determined by BAT or Calypso. BAT and Calypso localization data were summarized from 16236 imaging sessions and 1527 fractions, respectively.

### Results

The mean Calypso and BAT couch shifts in the lateral, vertical, and longitudinal directions were −1.3±5.6 mm, −4.3±6.4 mm, −0.7±3.8 mm and −0.1±11.4 mm, −1.3±11.6 mm, 0.5±12.8 mm, respectively. Both sets of data show that the largest localization error occurs in the posterior direction. However, with ultrasound‐based localization, patients are treated on average with isocenters 3.0 mm posterior to that determined by Calypso. Ultrasound localization distributions are 2 – 3 times wider than those of Calypso which is likely due to sources of uncertainty associated with BAT such as difficulty visualizing the prostate accurately and inter‐user variations.

### Conclusions

The Calypso system provides an objective method of localizing prostate patients. Relative to Calypso, ultrasound localization distributions suggest substantial inter‐user variability. In contrast, the Calypso system does not depend on the user's training or technique and provides objective information, potentially resulting in more accurate localization.

## A distributed‐computing approach to beam‐angle selection and dose optimization in IMRT

Daryl P. Nazareth, Matthew B. Podgorsak, Jubei Liu, Matthew D. Jones, and Michael R. Kuettel

Department of Radiation Medicine, Roswell Park Cancer Institute, Buffalo, NY; Center for Computational Research, University at Buffalo, Buffalo, NY

Commercial IMRT treatment planning software currently solves the dose optimization problem, but does not consider additional variables, such as gantry, collimator, or couch angles. These parameters are generally specified by clinicians based on their experience, and are often arbitrary and suboptimal. Including the additional variables in the treatment planning process increases the size of the combinatorial problem, and would require access to vast computational resources and novel optimization techniques. We have formed a collaboration with a leading academic supercomputer center and implemented the Nested Partitions (NP) algorithm, a versatile and efficient method, to produce IMRT plans containing optimal beam angles. NP is a powerful metaheuristic algorithm, which we employed in conjunction with the dose optimization algorithm of a commercial treatment planning system, making it well suited for distributed computing. We performed a study on a 5‐field prostate case by executing the programs on four machines simultaneously, and evaluated the resulting treatment plans using a scoring function based on DVH constraints for the target and critical structures. After only three iterations of the NP algorithm, the resulting plan realized an improvement of 14% over a clinical plan employing equi‐spaced beams. Since each iteration was implemented simultaneously on multiple machines, a decrease in execution time by a factor of about 4 was observed. Our results indicate that the use of a distributed‐computing platform combined with the NP algorithm can result in treatment plans which provide better target coverage and normal tissue sparing, and can be generated more efficiently than those currently produced.

## A Filmless Method to determine the accuracy of computed tomography (CT) gantry tilt indicators by Using Commercially Available Phantom

Ting‐Tung A. Chang, Cynthia M. Munoz, Mustapha R. Hatab, and Jonathan E. Tucker

Radiology, University of Texas Health Science Center at San Antonio, San Antonio, TX; Radiology, Brooke Army Medical Center, Ft Sam Houston, TX

### Purpose

To introduce a novel filmless method of performing CT gantry tilt angle QC to adapt the expanding digital radiology department.

### Method and Materials

A Catphan® 500 phantom was positioned according to the manufacturer's instructions in performing conventional CT QC. A specific slice thickness was chosen and image(s) obtained at a technique appropriate for the phantom. The true slice thickness $\left(T_{\text{slice}}\right)$ was measured by averaging the value obtained from all 4 ramps (using section 1, CTP401) following TG‐39 slice thickness protocol. The gantry was then tilted to an angle θ and the scans and FWHM length $\left(L_{\text{FWHM},\theta }\right)$ of the right wire ramp were measured. The value for $L_{\text{FWHM},\theta }$ was then inserted into the following formula: For $\theta >23,\;\theta =23+\cot^{-1}(L_{\text{FWHM},\theta }/T_{\text{slice}})$; for $23>\theta \geq -67,\;\theta =23-\cot^{-1}(L_{\text{FWHM},q}/T_{\text{slice}})$. The gantry angle calculated from these derived equations was validated by comparing with the angles from conventional film measurement method (TG‐39).

### Results

Different slice thicknesses obtained at 9 different angles from 16‐ and 64‐ slice scanners will be presented. On average, gantry tilt measured using the Catphan agreed with the conventional film measurement method within 1±.

### Conclusion

Our results confirm that actual gantry tilt can be determined within 1± consistently using the Catphan. This new method removes the requirement to use film for gantry tilt measurements. It is easy to calculate and can be performed without purchasing additional phantoms or equipment. The medical physicist can easily incorporate this into the scans obtained as part of the routine image quality testing.

## Monte Carlo simulation of complex IOHDR geometries

Moonseong Oh, Harish K. Malhotra, and Matthew B. Podgorsak

Department of Physics, State University of New York at Buffalo, Buffalo, NY 14260; Department of Radiation Medicine, Roswell Park Cancer Institute, Buffalo, NY 14263

The dose computation algorithm used to plan intra‐operative high dose rate (IOHDR) brachytherapy with commercial treatment planning systems (TPS) assumes a full scattering environment, and doses are delivered based on standard plans in which applicators are generally assumed to be flat because of the lack of imaging capabilities in the operating room. It has been shown that these approximations can lead to significant dose delivery errors. A few studies have reported this inaccuracy for general cases through either TPS calculations or experimental approaches. To quantify IOHDR dose errors more accurately, Monte Carlo calculations were performed to compare with measured known results for partial or full scattering treatment conditions, and also complex geometries (e.g., concave or convex structures with curved applicators). The setup geometry, including source structure, was designed with the geometry package using EGSnrc C++ library class. The user‐code was developed from an existing EGSnrc user‐code CAVRZnrc. Concave or convex applicator arrangements with six radii (range 1.35 cm to 12.5 cm) were used to simulate the treatment geometries, which were evaluated with 2‐dimensional plans. Monte Carlo results showed 1 % agreement with EDR2 film measurement for delivered doses except for very steeply curved structures on the convex side of curved applicators (about 2.5 % at 2 cm radius). This benchmarked Monte Carlo technique can be extended to any possible clinical geometry, thus improving dose calculations for complex clinical geometries and allowing the compilation of an accurate and comprehensive dosimetry atlas.

## The Aluminum Equivalence of PMMA under Computed Tomography Radiation Beam and Estimate of Scattering Distribution from CT Dose Measurement

Zhitong Yang, Robert Chu, and Chun Ruan

University of Oklahoma Health Sciences Center, Oklahoma City, OK 73126–0901

Aluminum (Al) has been the reference material for x‐ray absorbing and filtering properties. The thickness of different materials in relation to Al has been investigated in general diagnostic radiology. In this project, the Al equivalency of polymethyl methacrylate (PMMA), a very common tissue‐equivalent phantom material, is studied under the radiation beams of the computed tomography (CT) system used most in clinics of the United States. The results found with head filter and body filter are similar—a 10 mm PMMA filtration is roughly equal to 1.75 to 2.75 mm Al in the CT beam—over the energy range available on the CT scanner examined.

The filtration properties of PMMA are applied to CT dose measurements for both head and body phantoms. The direct beam contribution to dose is separated to estimate the axial scattering contribution. This estimation provided rough maps for the scattering dose distribution inside the phantom, and with respect to patient couch position.

### Conclusion

The established Al equivalency of PMMA under CT beam condition will help the effort in estimating CT HVL using CT dose measurement. The scatter distribution information could be useful for the estimation of noise level in developing algorithms for image reconstruction. Further studies may complement existing European literatures on the calculation of patient dose using different CT scanners.

## Pitfalls of the ITV and PTV dose in thoracic anatomy and composite 4D dose calculation

Jianzhou Wu, Raj Shekhar, and Warren D'Souza

Department of Radiation Oncology, University of Maryland School of Medicine, Baltimore, MD 21201; Department of Diagnostic Radiology, University of Maryland School of Medicine, Baltimore, MD 21201

Organs in the thorax and upper abdomen are susceptible to respiration induced internal organ motion. Due to organ motion, the dose delivered to tissues may differ from the planned dose. Four‐dimensional CT imaging and elastic image registration of the 4D CT images can be used in the delivered dose estimation. First, a treatment plan was generated on one of the CT image sets in the 4D CT. The plan parameters were copied to the remaining 3D CT images and the dose was recalculated on each individual image. Next, the recalculated doses were warped to the reference CT to form a composite 4D dose using transformation fields obtained from elastic image registration of the 4D CT. The composite 4D dose was treated as an accurate estimation of the dose delivered to the patient. In the literature, variation of the ITV and PTV doses corresponding to the different respiratory phases has been reported. In this study, we point out conceptual errors in the delivered dose estimation: first, the tissues constituting the ITV, and hence the PTV, in the context of mobile anatomy depends on the particular phase of the respiration cycle. Therefore the comparison of the ITV and PTV dose at different respiratory phases is erroneous. Its usage can easily lead to the incorrect calculations of the delivered dose to the patient. We therefore believe that while the ITV and PTV can be used to guide the addition of treatment margins, 4D dose calculations are only relevant to the GTV.

## Threshold for I131 Bioassay Monitoring in Nuclear Medicine

Yongguang Liang, Robert Y. Chu, Wendy K. Galbraith, George W. MacDurmon, and Jagadeesh R. Sonnad

The University of Oklahoma Health Sciences Center, 1100 N. Lindsay, Oklahoma City, OK 73104; Veterans Affair Medical Center, Oklahoma City OK 73104

Since the late nineteen seventies, manufacturers in nuclear medicine have reformulated the ^131^I solution to reduce the volatility of the iodine. There has also been an increase in use of the iodide in encapsulated form. The available results on the volatility of the reformulated radio‐iodine allow us to review the ^131^I bioassay program for nuclear medicine workers, which is required by the current US Nuclear Regulatory Commission (NRC) regulation (10 CFR 20.1502 2006). Our analysis shows the threshold quantity for ^131^I routine bioassay monitoring in nuclear medicine (444 GBq per quarter) far exceeds the general bioassay criteria set in the NRC Regulatory Guide 8.20 (1979). Furthermore, a single dose large enough to yield detectable thyroid burden (59.5 GBq dosage for a thyroid probe with MDA=925 Bq) is very unlikely to occur in a nuclear medicine clinic. Accidental ingestion or inhalation would be an exception to our conclusion.

